# Long QT Interval in Turner Syndrome – A High Prevalence of LQTS Gene Mutations

**DOI:** 10.1371/journal.pone.0069614

**Published:** 2013-07-25

**Authors:** Christian Trolle, Kristian H. Mortensen, Lisbeth N. Pedersen, Agnethe Berglund, Henrik K. Jensen, Niels H. Andersen, Claus H. Gravholt

**Affiliations:** 1 Department of Endocrinology and Internal Medicine and Medical Research Laboratories, Aarhus University Hospital, Aarhus, Denmark; 2 Department of Radiology, Cambridge University Hospitals, Cambridge, United Kingdom; 3 Department of Molecular Medicine, Aarhus University Hospital, Aarhus, Denmark; 4 Department of Cardiology, Aarhus University Hospital, Aarhus, Denmark; University of Tampere, Finland

## Abstract

**Objectives:**

QT-interval prolongation of unknown aetiology is common in Turner syndrome. This study set out to explore the presence of known long QT mutations in Turner syndrome and to examine the corrected QT-interval (QTc) over time and relate the findings to the Turner syndrome phenotype.

**Methods:**

Adult women with Turner syndrome (n = 88) were examined thrice and 68 age-matched healthy controls were examined once. QTc was measured by one blinded reader (intra-reader variability: 0.7%), and adjusted for influence of heart rate by Bazett’s (bQTc) and Hodges’s formula (hQTc). The prevalence of mutations in genes related to Long QT syndrome was determined in women with Turner syndrome and a QTc >432.0 milliseconds (ms). Echocardiographic assessment of aortic valve morphology, 24-hour blood pressures and blood samples were done.

**Results:**

The mean hQTc in women with Turner syndrome (414.0±25.5 ms) compared to controls (390.4±17.8 ms) was prolonged (p<0.001) and did not change over time (416.9±22.6 vs. 415.6±25.5 ms; p = 0.4). 45,X karyotype was associated with increased hQTc prolongation compared to other Turner syndrome karyotypes (418.2±24.8 vs. 407.6±25.5 ms; p = 0.055). In women with Turner syndrome and a bQTc >432 ms, 7 had mutations in major Long QT syndrome genes (SCN5A and KCNH2) and one in a minor Long QT syndrome gene (KCNE2).

**Conclusion:**

There is a high prevalence of mutations in the major LQTS genes in women with TS and prolonged QTc. It remains to be settled, whether these findings are related to the unexplained excess mortality in Turner women.

**Clinical Trial Registration:**

NCT00624949. https://register.clinicaltrials.gov/prs/app/action/SelectProtocol/sid/S0001FLI/selectaction/View/ts/3/uid/U000099E.

## Introduction

The corrected QT interval (QTc-interval) is prolonged in 33% of children and 20% of adults with Turner syndrome (TS) [1]–[3]. A prolonged QTc-interval is associated with increased risk of sudden death in the general population [4]. Long QT syndrome (LQTS) in general is characterized by prolongation of the QT-interval in association with the risk of Torsade de Point. LQTS comprises an acquired [5] as well as a congenital form [6]. However, in TS the mechanism behind the QTc prolongation is unknown. Women with TS characteristically display growth retardation with reduced final height and hypogonadism with infertility requiring hormone replacement therapy (HRT) [7]. Increased morbidity and mortality are present due to congenital heart disease, ischemic heart disease, hypertension, and diabetes [8]–[10]. A relative sinus tachycardia is a life-long phenomenon [1], [2], where conduction of the electrical impulse through the atria and atrioventricular node is accelerated [2], and the risk of atrial tachycardia may be increased [11] whereas bradycardia is rare [12]. Whether a QTc-interval prolongation contributes to the increased excess mortality in women with TS remains to be solved [9].

We therefore set out to examine the QTc-interval in women with TS assessing the prevalence of QTc prolongation and changes herein prospectively, with further view to assessing for the potential contribution of mutations in genes known to cause LQTS. The study aimed to relate findings to the phenotype, karyotype and the presence of cardiovascular malformations within the TS group. This study is the first to assess the QTc-interval in TS adhering to the recommendations of The American Heart Association using a linear function for QT adjustment of heart rate [13] and the first study to assess changes in the QTc-interval prospectively.

## Methods

### Ethics Statement

The study was approved by the Ethics Committee of the Central Denmark Region (#20010248) and registered at www.ClinicalTrials.gov (# NCT00624949). All participants gave written informed consent.

### Study Subjects

Women with karyotypically (Table 1) proven TS (*n* = 102) with a mean follow-up of 4.7±0.5 years were recruited through the Danish National Society of Turner Syndrome Contact Group and an endocrine outpatient clinic. The patients were examined at baseline, follow-up and end-of-study. Healthy, age-matched women (*n* = 68) were recruited by advertisement to serve as baseline controls. The controls were examined once.

**Table 1:** The karyotype distribution among woman with TS and an electrocardiogram.

Karyotype	Percent	hQTc >460 ms
45,X	60.2	4 of 53
Mosaic	39.8	
45,X/46,XX/47,XXX; 45,X/46,XX; 45,X/47,XXX	3.4	1 of 3
45,X/46,X,r(X);	4.5	
Turner Mosaic with Y-material	5.7	
Mosaic with isochromosom og dicentric chromosome	19.3	1 of 17
All other karyotypes	6.8	

### Electrocardiogram and QTc Measurements

Twelve lead Electrocardiograms (ECG) were obtained using the Personal 120/210 machine (Esaote Biomedica, Cambridge, United Kingdom). All ECGs were recorded at 25 mm/s with amplitude of 10 mm/mV. They were scanned to a digital file in 600 dpi and analyzed using Cardio Calipers 3.3 (Iconico, www.iconico.com) and measurements of RR and QT-interval were made on screen in lead II, V5 and V6. The U-wave was excluded using the “teach-the-tangent” method [13] and the bQTc-interval was calculated using Bazett’s formula and hQTc-interval using Hodges’s formula [14] in accordance with guidelines [13]. All ECGs were blinded and read by a single reader and the QTc established with an intra-observer variability of 0.7%. A bQTc-interval with duration of less than 460 milliseconds (ms) was defined as normal [13].

With respect to women with TS, ECG recordings were initiated at first follow-up. At this point 3 women with TS had died, 8 were lost to follow-up and 26 did not have an ECG taken or the ECG was of poor quality. Before end of study in total 4 women with TS had died, 14 were lost to follow-up and 8 did not have an ECG or the ECG was of poor quality. In order to gain the greatest power we included all women with TS who had an ECG taken at either visit 2 or 3 in a pooled TS group using the most recent ECG in the final analysis (n = 88). All controls had an ECG recording at baseline.

### Genetic Analysis

In women with TS and a bQTc >432 ms (N = 40) we determined the presence of mutations in genes related to LQTS (*KCNQ1, KCNH2, KCNE1, KCNE2, and SCN5A*). DNA was extracted from EDTA stabilized blood using Maxwell®16 Blood DNA Purification Kit. All coding exons including exon-intron boundaries of the genes KCNQ1, KCNH2, SCN5A, KCNE1, and KCNE2 were amplified and subsequently screened for mutations by High Resolution Melting Analysis using the LightScanner System (Idaho Technology Inc. Utah, USA), or by Sanger sequencing using a 3130XL Genetic Analyzer (Applied Biosystems). All primers for KCNQ1, KCNH2, KCNE1 and KCNE2 were designed in house. Primers for SCN5A were adapted from Millat [15], except for exons 2B, 5, 7, 10, 11, 12A, 13, 16, 17, 18, 20, 22, 27, and 28E which were designed in-house. Primer sequences and PCR conditions are available upon request. All samples were analyzed for large genomic deletions by Multiplex Ligation dependent Probe Amplification (MLPA) using the SALSA MLPA kit P114 Long-QT (MRC Holland, Amsterdam, Holland). Mutations were classified according to the “Classifications System for Sequence Variants”. [16].

### Laboratory Tests, Blood Pressures and Echocardiographic Assessment

Blood samples (sodium, potassium, calcium, triglyceride, low density lipoprotein and haemoglobin A1C), 24-hour blood pressures and echocardiography were performed at baseline, follow-up and end-of-study in women with TS and at baseline in controls.

Echocardiographic assessment of aortic valve morphology was done by one experienced observer on a GE Vivid Seven (GE Healthcare, Horten, Norway) with a 2.5 MHz transducer using second harmonic imaging. Images were obtained from parasternal and apical views during end-expiratory apnoea.

### Statistics

Statistical computations were performed using SPSS 20.0. Continuous variables were expressed as means±standard deviations or medians (range) as appropriate. Variables were computed as absolute, log-transformed or inverse-transformed values, and compared by Student’s independent t-test, Student’s paired t-test, Mann-Whitney U-test or Wilcoxon signed rank test as appropriate. For binary variables Fisher’s exact test was applied. Bivariate associations (r) of continuous variables were tested using Pearsońs coefficient of correlation, Spearman’s rank correlation coefficient or partial correlation coefficients. Further explanatory models were constructed for the primary outcome parameter, QTc-interval length, using backwards multiple linear regression analyses. Independent variables were chosen from the baseline correlation analyses of continuous variables and dichotomous variables (i.e. bicuspid aortic valve and karyotype). Independent variables were omitted from the models when P>0.10 and contributions to the final model were stated at standardized coefficients (β). Otherwise, P<0.05 was considered statistically significant.

The ECG findings are secondary outcomes from the original study assessing the aortic diameter by magnetic resonance imaging.

## Results

The QT-interval was prolonged in women with TS irrespective of correction method (Table 2). Ten (Bazett’s, 11% p = 0.005) and six (Hodges’s, 7% p = 0.036) of the 88 women with TS had a QTc ≥460 ms. None of the controls had a prolonged QTc. Twenty percent of women with TS had a bQTc and 15% a hQTc above 440 ms. There were no differences over time in hQTc in the 51 women with TS seen at both first follow-up and last visit (416.9±22.6 vs. 415.6±25.5; p = 0.4).

**Table 2:** Demographic, clinical and biochemical characteristics.

		Controls	Turner syndrome	P-value
		(n = 68)	(n = 88)	
Bazett's QTc	[ms]	389.1±20.1	426.8±30.1	<0.001
Hodges’s QTc	[ms]	390.4±17.8	414.0±25.5	<0.001
Heart rate	[bpm]	59.45±9,05	72.7±13.5	<0.001
Weight	[kg]	68.3±11.8	59.1±14.1	<0.001
Height	[cm]	168.4±6.23	147.0±7.10	<0.001
BMI	[kg/m]	22.7 (19.1–38.1)	26.0 (18.5–49.0)	<0.001
BSA	[m^2^]	1.76±0.16	1.51±0.18	<0.001
Age	[years]	38.9±12.4	42.3±10.4	0.07
24 h systolic BP	[mmHg]	112.7±10.5	117.4±13.3	0.02
24 h diastolic BP	[mmHg]	71.2±8.0	74.6±8.9	0.02
24 h heart rate	[bpm]	71.4±9.0	74.5±10.6	0.053
24 h heart rate daytime	[bpm]	74.7±9.2	77.6±11.0	0.09
24 h heart rate nighttime	[bpm]	62.3±8.6	67.8±10.5	0.001
Dipping	[%]	14.8±4.37	13.4±6.92	0.1
Hypertensive treatment	[%]	0±0	53.4±0.50	<0.001
HRT	[%]	0±0	81.8±0.39	<0.001
Statins prescribed	[%]	0±0	12.5±0.33	<0.003
Diabetes	[%]	0±0	12.5±0.33	<0.003
Calcium ion	[mmol/l]	1.22±0.04	1.21±0.04	0.057
Sodium	[mmol/l]	139.6±1.87	139.8±2.04	0.5
Potassium	[mmol/l]	3.73±0.23	3.77±0.26	0.3
LDL	[mmol/l]	2.88±0.85	2.90±0.77	0.9
Triglyceride	[mmol/l]	0.90 (0.4–4.5)	0.95 (0.4–2.6)	0.3
HbA1C	[%]	5.30 (4.6–6.6)	5.3 (4.5–8.6)	0.3

Values are expressed as mean±standard deviation or median (minimum–maximum).

BMI = body mass index, BSA = body surface area, BP = blood pressure, LDL = low density lipoprotein, HbA1C = Hemoglobin A1C, ms = milliseconds, bpm = beats per minute, NA = Not assessed, HRT = Hormone replacement therapy.

P-values<0.05 are considered statistically significant.

**Figure 1:**
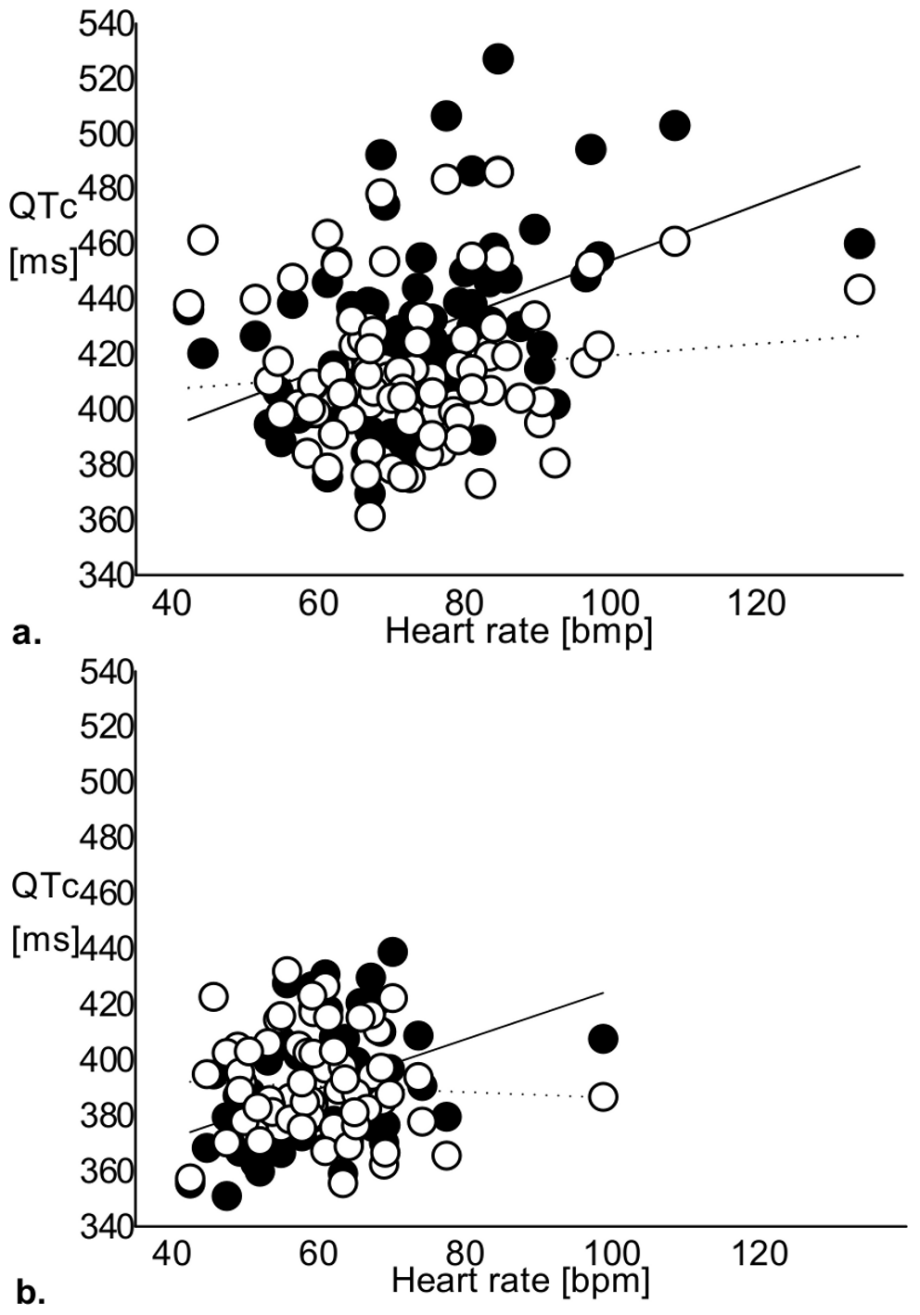
Heart rate plotted against QTc length. **a.** Heart rate plotted against QTc length in women with TS. Hodges’s formula (open circles, dashed regression line, r^2^ = 0.012; p = 0.3) effectively omitted the dependence of QTc on heart rate when compared to Bazett’s formula (filled circles, solid regression line, r^2^ = 0.200; p<0.001). **b.** Heart rate plotted against QTc length in controls. Hodges’s formula (open circles, dashed regression line, r^2^ = 0.003; p = 0.7) effectively omitted the dependence of QTc on heart rate when compared to Bazett’s formula (filled circles, solid regression line, r^2^ = 0.161; p = 0.001). ms = milliseconds, bpm = beats per minute.

Heart rate was plotted against QTc to clarify the contribution of heart rate to differences in the QTc-interval (Figure 1A and B). There was a positive correlation between heart rate and bQTc among women with TS (R = 0.448; p<0.001) and controls (R = 0.401; p<0.001), which was not present using hQTc (Figure 1A and B). Since the use of Hodges formula effectively removed the dependence of QTc on heart rate, we chose to use hQTc in all further calculations. A correlation was present between age and hQTc (r = 0.342; p = 0.004) in controls, but not in women with TS (Table 3).

**Table 3:** Bivariate correlations with hQTc.

		Controls		Turner syndrome	
		hQTc		hQTc	
Age	[years]	0.342	(0.004)	0,204	(0.06)
BMI	[kg/m]	0,206	(0.09)	0.089	(0.4)
Heart rate day time	[bpm]	−0.390	(0.001)	−0.142	(0.2)
Heart rate night time	[bpm]	−0.280	(0.02)	−0.053	(0.6)
Calcium Ion	[mmol/l]	−0.141	(0.3)	−0.232	(0.03)
Potassium	[mmol/l]	0.098	(0.4)	−0.192	(0.07)
Sodium	[mmol/l]	0.164	(0.2)	−0.269	(0.01)
Karyotype: Mosaic Vs.45,X				0.299	(0.03)

Values are expressed as Spearman’s Correlations coefficients (P-value).

BMI = body mass index, bpm = beats per minute.

P-values<0.05 are considered statistically significant.

### Karyotype and Molecular Genetics

The QTc was longer in women with TS and karyotype 45,X (Table 1) than all other karyotypes (hQTc 418.2±24.8 vs. 407.6±25.5; p = 0.055). In women with TS and a bQTc >432 ms (N = 40) we analyzed for the presence of Long QT (LQT) mutations, and found 8 with mutations in one or more of the analyzed genes. We found 6 with a mutation in the KCNH2 gene (five with c.3140G>T heterozygous p.Arg1047Leu, rs36210421; one with c.2738C>T heterozygous p.Ala913Val), two with a mutation in SCN5A (one with c.5872C>T p.Arg1958X; one with c.6010T>C heterozygous p.Phe2004Leu) (Table 4). One woman with TS who was found to have a mutation in the KCNH2 gene also had a KCNE2 gene mutation (c.161T>C heterozygous p.Met54Thr). The remaining 32 women with TS and the longest bQTc intervals did not have mutations in any of the studied genes. There was no difference neither in the bQT nor hQTc between those with and those without mutations (data not shown).

**Table 4:** Long QT mutation carriers’ descriptives.

Mutation	KCNH2^4^	KCNH2^1^	KCNH2^1^	KCNH2^1^	KCNH2^1^	SCN5A^2^	SCN5A^3^	KCNH2^1^
		KCNE2^5^						
De novo/inherited	Mater	?	Pater	?	?	Pater	Mater	?
Karyotype	45,X	45,X	45,X	45,X/46,X,i(Xq)	45,X,inv(9)	45,X	45,X/	45,X
				47,X,i(Xq),i(Xq)	(p11q11)		46,X,idic(X)	
Bazett's QTc [ms]*	468.7	465.2	464.8	458.3	453.5	453.4	443.4	432.0
Hodges's QTc [ms]*	444.3	433.9	435.8	434.6	427.6	452.6	424.8	415.6
Heart rate [bmp]	68	90	134	71	79	62	65	79
Bicuspid aortic valve	Yes	No	No	No	No	Yes	No	Yes
Coarctatio	No	No	Yes	No	No	Yes	No	No
Hypothyroidisme	No	Eltroxin	Eltroxin	No	No	Eltroxin	No	No
Ischemic heart disease	No	No	No	No	No	No	No	No
Diabetes mellitus	No	No	No	Yes	No	No	No	No
Hypertension	No	Yes	Yes	Yes	No	Yes	No	Yes
Antihypertensive drugs	No	Yes	Yes	Yes	Yes	Yes	Yes	No
Age [years]	26	56	43	42	32	41	38	38
HRT during entire study	No	Yes	Yes	Yes	Yes	Yes	Yes	Yes
BMI >30	No	Yes	Yes	Yes	No	Yes	Yes	No
Treatment for LQTS	No	No	?	β-blocker	β-blocker	No	?	β-blocker

* ECGs with the longest QTc-interval.

1 KCNH2 c.3140G>T heterozygous p.Arg1047Leu. Uncertain pathogenic, known mutation.

2 SCN5A c.6010T>C heterozygous p.Phe2004Leu. Uncertain pathogenic, known mutation.

3 SCN5A c.5872C>T heterozygous p.Arg1958X. Definitely pathogenic, Stop codon mutation.

4 KCNH2 c.2738C>T heterozygous p.Ala913Val. Definitely pathogenic.

5 KCNE2 c.161T>C heterozygous p.Met54Thr. Likely pathogenic.

A question mark indicates that no information was available. ms = milliseconds, bpm = beats per minute. HRT = Hormone replacement therapy.

The in silico analysis was perfomed by the use of Alamut software (Interactive Biosoftware, ver 2.1, Mont-Saint-Aignan, France, http://www.interactive-biosoftware.com/) taking into account results from Polyphen-2, SIFT, Grantham score, and the nucleotide and aminoacid conservation.

### Bicuspid Aortic Valve and QTc

The presence or absence of bicuspid aortic valve was not associated with hQTc prolongation in women with TS (418.6±23.9 vs. 411.7±26.0; p = 0.3).

### QTc Correlations and Multiple Regression Analysis

We then went on to study the dependent variable hQTc in backward multiple linear regression analyses, including variables that had proven to be predictors of hQTc in bivariate correlation analyses (Table 3) and which theoretically could have an effect on the QTc-interval. In one such model daytime heart rate (β = −0.001; p<0.001), calcium ion (β = −0.125; p = 0.059), potassium (β = −0.023; p = 0.01), sodium (β = −0.004; p = 0.01), BMI (β = 0.001; p = 0.02), and age (β = 0.0005; p<0.001) were independent explanatory variables of hQTc in women with TS. Variables obtained from medicinal care records, 24-hour blood pressures and other serum markers did not prove to be of use. In controls explanatory variables of hQTc were age (β = 0.001; p<0.001), daytime heart rate (β = −0.001; p<0.001), and calcium ion (β = −0.093; p = 0.052).

As mentioned four women with TS died prior to having an ECG taken for the purpose of the study. One died following operation of an aortic aneurism (hQTc = 428.8 ms), one due to non-witnessed epileptic seizure (hQTc = 435.2 ms), one from unexpected cardiac arrest 5 days after a colon resection (hQTc = 402.7 ms) and the last was found dead in her home (no ECG the patient file).

## Discussion

The principal results from our study provide evidence that mutations in LQTS genes are highly prevalent in adult women with TS and a QTc >432 ms. We speculate that these findings may be one of numerous possible mechanisms behind QTc prolongation in TS. However, this study does not answer the question as to what extent mutations in LQTS genes may explain a proportion of the unexplained excess mortality which is well known in Turner cohorts [9], [10].

Moreover, we assessed the QTc-interval prospectively since a prolongation of the QTc-interval over time could suggest acquired LQT due to e.g. medication prescribed to treat comorbidities. However the QTc-interval did not change during the follow-up period.

Previous studies of QTc in TS have used Bazett’s formula which retains a strong residual correlation with heart rate [13], as shown here. According to guidelines [13] we estimated QTc using a linear approach (Hodges’s formula) as well as Bazett’s formula, and results depend on the method used. Our use of both equations makes comparisons with previous studies feasible and Hodges’s formula effectively eliminates any residual correlation with heart rate. We found the prevalence of QTc above 440 ms (bQTc 20% and hQTc 15%) comparable with previous studies (21–36%) [1]–[3]. We suggest future use of Hodges’s formula when estimating QTc as to remove the dependence of heart rate.

In the general population 4% of Caucasians may have a LQTS positive gene test [6], which markedly contrasts with the prevalence of 20% of women with TS in the genetically screened group resulting in a prevalence of at least 9% in the complete TS cohort. In the TS cohort 7.5% of the mutations where known pathogenic mutations. We have no genetic explanation for the apparent co-segregation of LQT mutations with the complete or partial loss of an X in TS.

Seven of the 8 LQT mutations in the present study were missense mutations and one resulted in a stop codon. KCNH2 c.3140G>T is often reported as a polymorphism without detectable differences in channel function when compared to the wild type [17], [18] though not consistently [19]–[21]. A prevalence of 4% has been reported in a Danish cohort [21]. It has been speculated that KCNH2 c.3140G>T may be associated with particular susceptibility towards hypokalemia or certain drugs, as it seems the case in hypokalemic patients with C-terminal mutations in HERG [22]. Lastly, it has been reported in patients with Torsade des Pointes ventricular tachycardia [23]. The SCN5A c.5872C>T results in a stop codon mutation. The SCN5A c.6010T>C mutation has been linked to sudden infant death [24] and increased persistent sodium current [24], [25]. In addition, depolarizing shifts in voltage dependence of inactivation and faster recovery from inactivation [25] have been described. KCNE2 c.161T>C has been related to exercise induced ventricular tachycardia [26], Torsade des Pointes ventricular tachycardia [27], diminished potassium flux, a less readily activation and a more rapid deactivation. The latter gene mutation has also been encountered in procainamide-induced LQTS and carriers of this mutation have longer QTc-intervals. [28] KCHN2 c.2738C>T has been reported to lead to the Romano-Ward syndrome [29], [30]. Considering the in silico prediction results the mutations were classified as shown in Table 4.

Our study confirmed a negative correlation with ionized calcium and potassium but also sodium in TS but opposed to the healthy controls. Interestingly, mean values of calcium, sodium and potassium were comparable with controls and within normal limits. Calcium was the only variable with an impact on the hQTc-interval in our multiple regression analysis. Prior studies have explored differences in electrolytes between women with TS and controls as well as groups with TS and prolonged versus normal QTc. However, an actual correlation between the electrolytes and the QTc is a novel finding [1]–[3], [31]. A correlation between QTc and calcium within the reference interval has been described in non-TS patients with cirrhosis [32] and with serum sodium [33]. Whether repolarization in women with TS (and thus the resultant QTc-interval) is more sensitive to changes in electrolytes within the normal range is not known.

The aetiology of the altered cardiac electrophysiology in TS is unknown but several aspects suggest an inherited defect. In accordance with Dalla Pozza et al [3], but in contrast to others [1], [2], we found the 45,X karyotype to have an adverse impact on the heart rate corrected QT-interval. Secondly the hQTc-interval prolongation is not associated with traditional indices of QTc-interval elongation (i.e. age, left ventricular hypertrophy, medicinal use, coronary heart disease and thyroid disorders) [1], [2]. Bicuspid aortic valve had no influence on hQTc, a finding confirmed by previous studies. [1], [2].

Gender affects QTc as is evident through testosterone mediated shortening of QTc-intervals in males [34], [35]. Endogenous as well as exogenous progesterone also shorten QTc [36] and counteracts a QTc prolonging effect of exogenous estrogen [35], [36] and therefore HRT does not affect QTc [37]. Low endogenous progesterone and androgens as well as unopposed estrogen therapy could therefore in women with TS, at least on a theoretical level, prolong QTc-intervals.

The length of the QTc-interval in women with TS differs from that of age matched girls with short stature. [3] It remains to be resolved, if the X-chromosomal haploinsufficiency in women with TS results in altered transcription of proteins involved in the activity of ion channels as structural or regulatory components, such as is seen in congenital LQTS [38]. Alternatively, altered expression of X-linked genes may function as transcriptional regulators of autosomal genes involved in ion channel activity. Interestingly, the action potential of cardiomyocyte-like differentiated pluripotent stem cells is prolonged in women with TS [39], which points towards a primary developmental defect. Our multiple regression analysis explained less than 30% of the variation in the hQTc-interval length. Further insight is necessary into the aetiology of QTc prolongation, including the role played by the relatively increased sympathetic drive in women with TS [40], [41] that likely increases automaticity, excitability and conductivity of cardiomyocytes and cardiac conduction tissues.

Drug therapy, including HRT, antihypertensive and cholesterol lowering drugs was not correlated with hQTc in this cohort. Interestingly, the empiric recommendation of beta-blockers to treat aortic dilatation [42] may have been beneficial in numerous cases, since beta-blockers are the drug of choice in patients with TS [43].

### Limitations

Since differing height and body composition is inherent in women with TS we included BMI and BSA as a confounder in our calculations instead of choosing a control group matching the former mentioned criteria since such controls cannot be considered entirely healthy.

No information on the plasma levels of sex hormones was available. Yet it can be noted that no difference was demonstrated between patients with or without HRT.

We screened women with TS and a bQTc >432.0 ms and no controls. Hence there is the possibility that the controls harbour the same prevalence of mutations or that the women with TS who were not screened for mutations had a different prevalence of LQT mutations.

### Future Perspectives

We anticipate that future studies will provide more knowledge about the genetic and epigenetic profile of women with TS, aspects of inheritance as well as the effect of androgens and progesterone. Use of Hodges’s formula to estimate the QTc should be recommended to eliminate the influence of heart rate. In the clinical care of women with TS, the clinician should be aware of LQTS. Annual ECGs and on a regularly basis evaluation by a cardiologist is paramount. Beta-blockers may be prescribed as first choice antihypertensive drug targeting hypertension as well as LQTS. Care should be taken to avoid drugs known to lengthen the QTc-interval and if long-term unopposed estrogen therapy is prescribed, it should be followed by regular reevaluation of the QTc-interval.

### Conclusion

There is a high prevalence of mutations in the major LQTS genes in women with TS and prolonged QTc. However, it remains to be settled, whether these findings are related to the unexplained excess mortality in Turner women.
